# Tocilizumab and COVID-19: a meta-analysis of 2120 patients with severe disease and implications for clinical trial methodologies

**DOI:** 10.3906/sag-2010-131

**Published:** 2021-06-28

**Authors:** Azza SARFRAZ, Zouina SARFRAZ, Muzna SARFRAZ, Hinna AFTAB, Zainab PERVAIZ

**Affiliations:** 1 Department of Pediatrics and Child Health, Aga Khan University, Karachi Pakistan; 2 Research Department, Fatima Jinnah Medical University, Lahore Pakistan; 3 Department of Physiology, CMH Lahore Medical and Dental College, Lahore Pakistan; 4 Research Department CMH Lahore Medical and Dental College, Lahore Pakistan

**Keywords:** Tocilizumab, actemra, coronavirus, COVID-19, clinical trial, cytokine storm syndrome

## Abstract

**Background/aim:**

Since the outbreak of the COVID-19, numerous therapies to counteract this severe disease have emerged. The benefits of Tocilizumab for severely infected COVID-19 patients and the methodologies of ongoing clinical trials are explored.

**Materials and methods:**

A systematic search adhering to PRISMA guidelines was conducted in PubMed, Cochrane Central, medRxiv, and bioRxiv using the following keywords: “Tocilizumab,” “Actemra,” “COVID-19.” An additional subsearch was conducted on Clinicaltrials.gov to locate ongoing tocilizumab trials.

**Results:**

A total of 13 studies were included in the meta-analysis comprising 2120 patients. The treatment group had lower mortality compared to the control group (OR = 0.42, 95% CI = 0.26 to 0.69, P = 0.0005, I2 = 55%). A descriptive analysis of 50 registered trials was conducted.

**Conclusion:**

This review meta-analyzed the therapeutic benefits of tocilizumab in COVID-19 patients with severe disease for mortality, mechanical ventilation, and the characteristics of COVID-19 registered trials.

## 1. Introduction 

Since the outbreak of the novel coronavirus disease 2019 in December 2019, throughout the Hubei province of China, several clinical trials have been conducted to assess the benefits of certain therapies. Tocilizumab, also known as atlizumab, is an immunosuppressive drug, mainly for the treatment of rheumatoid arthritis and systemic juvenile idiopathic arthritis, a severe form of arthritis in children. It is a humanized monoclonal antibody against the interleukin-6 receptor. Although every observational study of COVID 19 so far has hinted at the benefits of drugs that may block inflammatory cytokines, trials of interleukin 6 inhibitors, such as sarilumab, have yet to show any viable benefits [1]. As of 13th October 2020, 1.08 million individuals have died due to coronavirus disease 2019 (COVID-19), with 37.6 million cases documented worldwide. While the death toll reaching over 1 million individuals worldwide, the results of randomized, double-blind, placebo-controlled trials have raised questions about the benefits or tocilizumab in patients with COVID-19 [2]. 

The World Health Organization (WHO) estimates that the mortality rate of disease caused by COVID-19 is 3.7%, which is 10 times higher than that seen in influenza [3]. Afflicted patients may have an overwhelming immune reaction causing the cytokine storm syndrome with elements of the Macrophage Activation Syndrome (MAS), Cytokine-Release Syndrome (CRS), leading to Acute Respiratory Distress Syndrome (ARDS). SARS-CoV-2 leads to the production of inflammatory cytokines including Interleukin-6, which then contributes to cytokine storm syndromes damaging the lungs and other organs, ultimately leading to death. IL-6 is a pleiotropic proinflammatory cytokine produced by many cell types including fibroblasts, monocytes, and lymphocytes. The SARS-CoV-2 infection leads to a dose-based production of IL-6 from bronchial cells [4]. Interleukin inhibitors may help ameliorate severe damage to the lung tissue caused by cytokine release in patients infected with severe COVID-19 disease.

As of September 2020, there are 280 COVID-19 clinical trials registered for the treatment of COVID-19 in clinicaltrials.gov. The clinical course and mortality outcomes have baffled the population and healthcare organizations due to the heterogeneity in clinical presentations with some being asymptomatic to others acquiring severe pneumonia with respiratory failure leading to mechanical ventilation or death [5]. 

While the National Institute of Health guidelines proposed that insufficient data were present to support the use of interleukin-6 inhibitors other than for COVID-19 clinical trials, until more concrete evidence is available, healthcare providers must exercise caution in prescribing immune-modulating therapies [6]. This meta-analysis reviews the benefits of Tocilizumab for severe COVID-19 patients and critiques the methodologies of ongoing clinical trials.

## 2. Materials and methods

### 2.1. Search strategy 

All potential studies were identified by conducting a systematic search using PRISMA guidelines. Two databases were searched to include observational studies including PubMed (MEDLINE) and Cochrane Central. Additional studies were located using medRxiv and bioRxiv, in addition to grey literature sources, such as the WHO-COVID database and Google Scholar. A combination of keywords was used including “Tocilizumab,” “Actemra”, and “COVID-19.” An additional search was conducted on Clinicaltrials.gov to locate all ongoing tocilizumab trials.

### 2.2. Inclusion and exclusion criteria 

All studies comparing the clinical benefits and all-cause mortality outcomes of tocilizumab were included in the study. The comparators for the treatment for COVID-19 named as controls in our study were receiving standard treatment of care or no treatment. Articles published after January 1st, 2020, were included with no language restrictions. We excluded 1) case studies, 2) case series, 3) letter to editors, 4) single-arm studies, and 5) two-arm studies that did not report any outcomes of interest. 

6. National Institutes of Health (2020). COVID-19 Treatment Guidelines Panel. Therapeutic Options Under Investigation, Coronavirus Disease COVID-19 [online]. Website https://www.covid19treatmentguidelines.nih.gov/ [accessed 20 September 2020].

Two early-to-mid level researchers (ZS and AS) searched the articles independently. All discrepancies were resolved by the third author (MA). Data were systematically collected for the primary outcome, which was mortality in both treatment and control group, and the secondary outcome, mechanical ventilation using a shared spreadsheet for the meta-analysis. For all ongoing clinical trial registrations, findings were tabulated as 1) clinical trial identifier, 2) study design, 3) estimated enrollment, 4) conditions, 5) phase of the study, 6) interventions (experimental vs. comparator), 7) primary outcome measure and 8) recruitment status.

### 2.3. Objectives

The primary objective was to determine whether tocilizumab reduced the risk of mortality in the treatment groups. The secondary objective was to identify if the risk of mechanical ventilation was higher in either group. 

### 2.4. Data analysis 

All analyses were carried using Review Manager V5.4. The Mantel–Haenszel random-effects model was used with 95% confidence intervals. A test of P ≤ 0.05 was considered significant. A presentation of the Unadjusted Odds ratios (ORs) was given for dichotomous variables (I. mortality and II. mechanical ventilation). The I2 index was identified to assess heterogeneity among the included studies. A funnel plot was generated for visual inspection if more than 10 studies were included in either analysis. The study was not registered in an online register due to the time-sensitivity of the topic. 

## 3. Results

**Figure 1:**
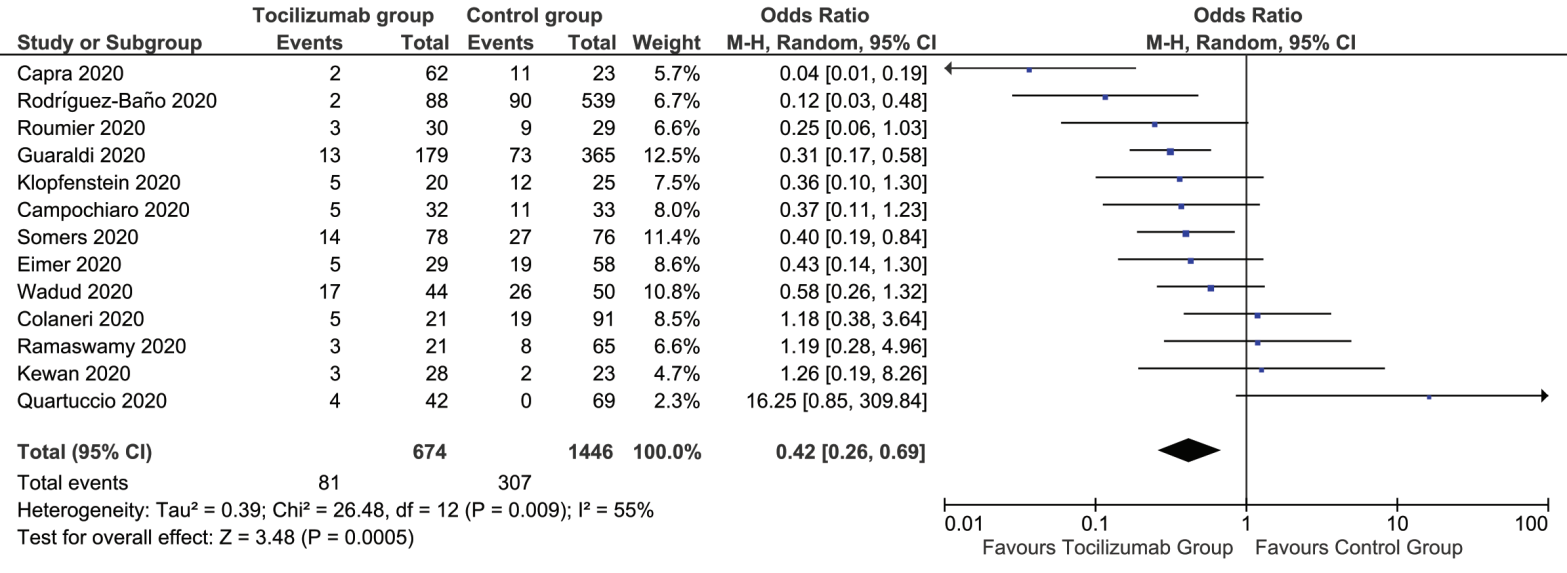
Mortality associations between the tocilizumab group and the control group.

**Figure 2:**
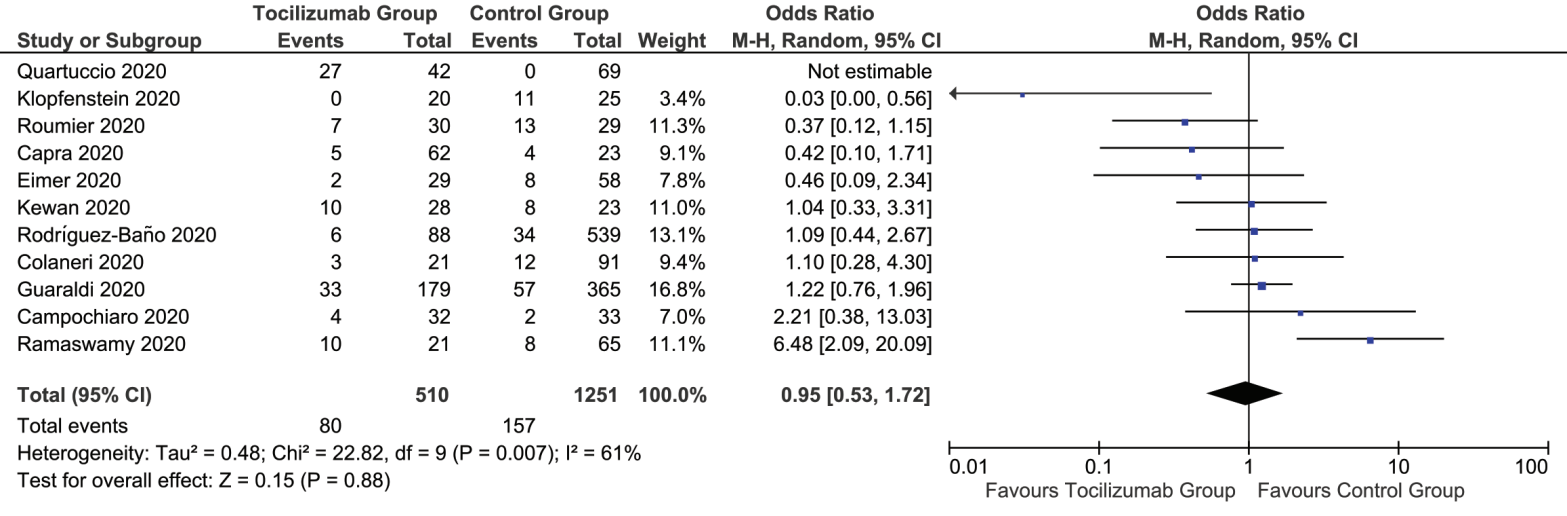
Odds of mechanical ventilation between the tocilizumab group and the control group.

**Figure 3:**
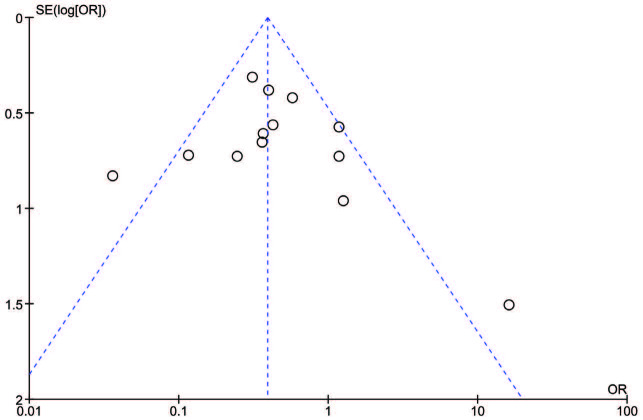
Funnel plot showing publication bias of included studies. Each dot represents a single study. The X axis shows the result of the study expressed as an odds ratio for mortality in tocilizumab and control groups. The Y axis is the standard error of the effect estimate. The shape is asymmetrical as it does not resemble that of an inverted funnel or a pyramid representing publication bias. Given that the I^2^ value was 55%, study heterogeneity may have led to an asymmetrical funnel.

We included 13 studies in the meta-analysis [5,7–17]. There were a total of 2120 patients, with 674 (31.8%) patients in the tocilizumab group and 1446 (68.2%) patients in the control group. Our results indicate that patients treated with tocilizumab had lower risks of mortality as compared to those who received no treatment or standard COVID-19 therapies (OR = 0.42, 95% CI = 0.26 to 0.69, P = 0.0005). There was moderate heterogeneity in included studies (I2 = 55%) (Figure 1), whereas, the risk of mechanical ventilation was inconclusive in those who obtained tocilizumab therapy (OR = 0.95, 95% CI = 0.53 to 1.72, P = 0.88, I2 = 61%) (Figure 2). The included studies provide clear evidence that the treatment group has a lower risk of death in severe COVID-19 patients, requiring further proof in the form of randomized controlled trials. However, the findings do not identify a clear demarcation of risks of mechanical ventilation between the treatment and control groups. Publication bias was noted within acceptable limits and high to moderate level methodological studies were included in our analysis (Figure 3).

Using the keywords “COVID-19” and “tocilizumab,” a total of 67 studies were located on Clinicaltrials.gov, of which we present a total of 50 registered trails. Of all, we included 4(8%) nonrandomized, open-label studies, 7(14%) randomized, double-masked (participant and investigator) studies, 30(60%) randomized, open-label studies, 1(2%) randomized, quadruple masking (participant, care provider, investigator, outcomes assessor) study, 1(2%) randomized, single masking (investigator) study, and 7(14%) single group, open-label studies (Table). 

**Table:** Characteristics of registered tocilizumab trials.

No	Clinical trialidentifier	Study designs	EstimatedEnrollment	Current phase(Updated 9/14/2020)	Interventions	Primary outcome measures	Recruitment status
1	NCT04331795	Nonrandomized, open label	32	Phase 2	Tocilizumab	Clinical response	Completed
2	NCT04339712	Nonrandomized, open label	40	Phase 2	Anakinra, Tocilizumab	Change of baseline total sequential organ failure assessment (SOFA) score|	Recruiting
3	NCT04492501	Nonrandomized, open label	600	Not Applicable	Therapeutic plasma exchange, convalescent plasma, tocilizumab, remdesivir, Mesenchymal stem cell therapy	Survival	Completed
4	NCT04423042	Non-Randomized, open label	30	Phase 3	Tocilizumab	All-cause mortality	Not yet recruiting
5	NCT04438980	Randomized, double masking between participant and investigator	72	Phase 3	Methylprednisolone vs. Placebo	Proportion of patients developing treatment failure and Mortality at day 28	Recruiting
6	NCT04412772	Randomized, double masking between participant and investigator	300	Phase 3	Tocilizumab vs. Placebo	Clinical status (on a 7-point ordinal scale) at day 28	Recruiting
7	NCT04356937	Randomized, double masking between participant and investigator	243	Phase 3	Tocilizumab vs. Placebo	The time from administration of the investigational agent (or placebo) to requiring mechanical ventilation and intubation, or death for subjects who die prior to intubation|	Active, not recruiting
8	NCT04372186	Randomized, double masking between participant and investigator	379	Phase 3	Tocilizumab vs. Placebo	Cumulative Proportion of ParticipantsRequiring Mechanical Ventilation by Day 28	Active, not recruiting
9	NCT04320615	Randomized, double masking between participant and investigator	450	Phase 3	Tocilizumab vs. Placebo	Clinical Status Assessed Using a 7-Category Ordinal Scale	Completed
10	NCT04409262	Randomized, double masking between participant and investigator	450	Phase 3	Remdesivir, Tocilizumab vs. Placebo	Clinical Status as Assessed by the Investigator Using a 7-Category Ordinal Scale of Clinical Status on Day 28	Recruiting
11	NCT04380519	Randomized, double masking between participant and investigator	372	Phase 2|Phase 3	RPH-104 80 mg, Olokizumab 64 mg; vs. Placebo	Proportion of patients, responded to the study therapy, in each of the treatment groups	Recruiting
12	NCT04479358	Randomized, open label	332	Phase 2	Tocilizumab vs. Standard of Care	Time to Recovery, Achievement of Recovery and Overall Survival	Not yet recruiting
13	NCT04345445	Randomized, open label	310	Phase 3	Tocilizumab, Methylprednisolone	The proportion of patients requiring mechanical ventilation	Not yet recruiting
14	NCT04435717	Randomized, open label	78	Phase 2	Tocilizumab 20 MG/ML Intravenous Solution;Drug: Tocilizumab 20 MG/ML Intravenous Solution (2 doses)	Change in IL-12 values in the 3 study groups from the start of treatment (Day 0) and on days 1 and 3	Recruiting
15	NCT04377750	Randomized, open label	500	Phase 4	Tocilizumab	Survival	Recruiting
16	NCT04332094	Randomized, open label	276	Phase 2	Tocilizumab, Hydroxychloroquine, Azithromycin	In-hospital mortality	Recruiting
17	NCT04377659	Randomized, open label	40	Phase 2	Tocilizumab	Progression of respiratory failure or death	Recruiting
18	NCT04412291	Randomized, open label	120	Phase 2	Anakinra Prefilled Syringe, Tocilizumab Prefilled Syringe vs. Standard-of-care treatment	Time to recovery and Mortality	Recruiting
19	NCT04346355	Randomized, open label	126	Phase 2	Tocilizumab	Entry into Intensive Care with invasive mechanical ventilation or death from any cause or clinical aggravation	Terminated
20	NCT04377503	Randomized, open label	40	Phase 2	Tocilizumab 180 MG/ML, Methylprednisolone Sodium Succinate	Patient clinical status 15 days afterrandomization	Not yet recruiting
21	NCT04363736	Randomized, open label	100	Phase 2	Tociliuzumab	Serum Concentration of interleukin-6 (IL-6) Following Administration of 8 mg/kg IV TCZ	Completed
22	NCT04361032	Randomized, open label	260	Phase 3	Tocilizumab Injection, Deferoxamine	Mortality rate	Not yet recruiting
23	NCT04424056	Randomized, open label	216	Phase 3	Anakinra, Ruxolitinib vs. Standard of care	Ventilation free days at day 28	Not yet recruiting
24	NCT04403685	Randomized, open label	129	Phase 3	Tocilizumab	Evaluation of clinical status and All-cause mortality	Terminated
25	NCT04335305	Randomized, open label	24	Phase 2	Tocilizumab, Pembrolizumab (MK-3475)	Percentage of patients with normalization of SpO2 on room air (measured without any respiratory support for at least 15 minutes) and Proportion of patients discharged from the emergency department and classified as low risk	Recruiting
26	NCT04333914	Randomized, open label	384	Phase 2	Chloroquine analog (GNS651), Nivolumab, Tocilizumab, Standard of care, Avdoralimab, Monalizumab	28-day survival rate and Time to clinical improvement	Suspended
27	NCT04476979	Randomized, open label	120	Phase 2	Tocilizumab, Dexamethasone	Survival without needs of ventilator utilization at day 14 and WHO progression scale at day 7 and 14	Recruiting
28	NCT04361552	Randomized, open label	0	Phase 3	Best Practice, Tocilizumab	7-day length of invasive mechanical ventilation (MV)	Withdrawn
29	NCT04330638	Randomized, open label	342	Phase 3	Usual Care, Anakinra, Siltuximab, Tocilizumab	Time to Clinical Improvement|	Recruiting
30	NCT04331808	Randomized, open label	228	Phase 2	Tocilizumab	Survival without needs of ventilator utilization at day 14. Group 1	Active, not recruiting
31	NCT04322773	Randomized, open label	200	Phase 2	RoActemra IV vs. Standard medical care	Time to independence from supplementary oxygen therapy	Recruiting
32	NCT04381936	Randomized, open label	15000	Phase 2|Phase 3	Lopinavir-Ritonavir, Corticosteroid, Hydroxychloroquine, Azithromycin, Convalescent plasma, Tocilizumab, Immunoglobulin	All-cause mortality and Duration of hospital stay	Recruiting
33	NCT04536363	Randomized, open label	284	Phase 2	Analogs, Prostaglandin vs. Standard therapeutic protocol	Mortality and Hypoxemia Resolution	Not yet recruiting
34	NCT04359095	Randomized, open label	1600	Phase 2|Phase 3	Hydroxychloroquine|Drug, Lopinavir / Ritonavir Pill, Azithromycin, vs. Standard treatment	Mortality	Not yet recruiting
35	NCT02735707	Randomized, open label	7100	Phase 4	Fixed-duration Hydrocortisone, Shock-dependent hydrocortisone, Ceftriaxone, Moxifloxacin or Levofloxacin, Piperacillin-tazobactam, Ceftaroline, Amoxicillin-clavulanate, Macrolide administered for 3-5 days or 14 days, 5/10-days oseltamivir, Lopinavir/ritonavir, Hydroxychloroquine Hydroxychloroquine + lopinavir/ritonavir|Drug: Interferon-1a Anakinra, Fixed-duration higher dose Hydrocortisone, Tocilizumab, Sarilumab, Vitamin C, Therapeutic anticoagulation, Simvastatin, Convalescent plasm,Protocolised mechanical ventilation strategy	All-cause mortality	Recruiting
36	NCT04374539	Randomized, open label	116	Phase 2	Plasma exchange vs. Standard medical treatment	Impact of plasma exchange	Recruiting
37	NCT04366245	Randomized, open label	72	Phase 1|Phase 2	Hyperimmune plasma vs Standard of care for SARS-CoV-2 infection	Safety: Incidence of Adverse Events and Serious Adverse Events grade 3 and 4, related to the product under investigation or the administration procedure, graduated according to the common toxicity criteria scale (CTCAE)	Recruiting
38	NCT04346693	Randomized, open label	320	Phase 3	Standard therapy recommended by the Ministry of Health of the Russian Federation and Dalargin intramuscular injection	The change of viral load in patients with SARS-COVID-19	Active, not recruiting
39	NCT04401410	Randomized, open label	58	Phase 1	Dose Finding Phase (MTD), Partially HLA-matched SARS-CoVSTs, Routine care	Graft versus Host Disease (GvHD) and Cytokine Release Syndrome (CRS)	Not yet recruiting
40	NCT04392414	Randomized, open label	60	Phase 2	COVID-19 convalescent hyperimmune plasma, Non-convalescent fresh frozen plasma (Standard plasma)	30-day mortality rate	Recruiting
41	NCT04414631	Randomized, open label	120	Phase 2	Conestat alfa	Disease severity and Time to clinical improvement	Recruiting
42	NCT04335071	Randomized, quadruple masking among participant, care provider, investigator, and outcomes assessor	100	Phase 2	Tocilizumab, Placebo	Number of patients with ICU admission, intubation and death	Recruiting
43	NCT04349410	Randomized, singlemasking for investigator	500	Phase 2|Phase 3	Hydroxychloroquine, Azithromycin/ Doxycycline/ Clindamycin/ Primaquine - low dose, Clindamycin,Primaquine - high dose, Remdesivir, Tocilizumab, Methylprednisolone, Interferon-Alpha2B, Losartan, Convalescent Serum	Improvement in FMTVDM Measurement with nuclear imaging, Ventilator status, and survival status	Enrolling by invitation
44	NCT04445272	Single group, open label	500	Phase 2	Tocilizumab	Mortality rate and metrics of respiratory function	Recruiting
45	NCT04317092	Single Group, open label	400	Phase 2	Tocilizumab Injection	One-month mortality rate	Recruiting
46	NCT04363853	Single Group, open label	200	Phase 2	Tocilizumab	Hematic biometry and Blood chemistry	Recruiting
47	NCT04370834	Single group, open label	217	Phase 2	Tocilizumab	Clinical outcome as evaluated by the 7-category Clinical Status Ordinal Scale	Suspended
48	NCT04315480	Single group, open label	38	Phase 2	Tocilizumab	arrest in deterioration of pulmonary function and death	Active, not recruiting
49	NCT04386239	Single Group, open label	40	Early Phase 1	Sarilumab Prefilled Syringe	Proportion of patients who show an improvement of the respiratory function	Not yet recruiting
50	NCT04335123	Single Group, open label	50	Phase 1	Losartan	Number of participants with treatment-related adverse events as assessed by protocol definition of AE	Recruiting

## 4. Discussion

To the best of our knowledge, this is the first review that meta-analyses the benefits of tocilizumab in severe COVID-19 patients along with reviewing methodologies of ongoing clinical trials. Our findings must be read with caution due to the lack of strong evidence from randomized controlled trials. Tocilizumab is the humanized anti-IL-6 receptor monoclonal antibody that is approved specifically for cytokine release syndrome, systematic juvenile idiopathic arthritis, and rheumatic arthritis [18]. The results indicate that tocilizumab improves mortality outcomes in severely infected COVID-19 patients; however, the results do not elucidate clear demarcation of risks of mechanical ventilation between treatment and control groups.

The National Institute of Health published Interim guidelines suggesting that tocilizumab may be considered for COVID-19 patients meeting the following six criteria, who are otherwise ineligible for steroid therapy [19]. The patient 1) must be COVID-19 positive, 2) must have abnormal chest imaging as seen in coronavirus infections, worsening respiratory status over 1–2 days necessitating 4–6 L/min of oxygen, 3) must not have any systemic fungal or bacterial co-infection, 4) must be suspected for cytokine release syndrome support by an elevation of inflammatory markers (for example, D-dimer > 1 mg/L, Ferritin > 600 ug/mL, LDH > 250U/L), along with clinical decline, 5) must not have had a poor prognosis indicating an unlikely survival of over 48 h, and 6) must have received mechanical ventilation for 24 h or less. 

Of the studies included in the meta-analysis, Somers et al. only tested the benefits of tocilizumab on severe COVID-19 patients obtaining mechanical ventilation. The duration of mechanical ventilation among the tocilizumab and control group was 13.8d (IQR: 7.1d, 27.5d) and 13d (IQR: 8.1d, 23.5d), respectively [12]. It is noted that the benefits of tocilizumab therapy on patients who have been mechanically ventilated patients for over 24 h may be low due to low chances of clinical improvement. 

19. Centers for Disease Control and Prevention (2020). Information for Clinicians on Investigational Therapeutics for Patients with COVID-19 [online]. Website https://www.cdc.gov/coronavirus/2019-ncov/hcp/therapeutic-options.html [accessed 20 September 2020].

However, 56% of patients (n = 76) in the treatment group were discharged alive by the end of follow-up process as compared to 30% of patients (n = 76) in the control group suggesting that the clinical benefits must be assessed in the ongoing placebo-controlled trials. In the COVACTA trial, where 452 severe COVID-19 patients were randomized, improvements in clinical status at day 28 and mortality outcomes were not observed [20]. Both the safety and efficacy of tocilizumab must be assessed for patients with severe disease in the several randomized, double-blind, placebo-controlled phase 3 trials namely COVACTA, REMDACTA, and EMPACTA to corroborate the true benefits of the drug in acute care settings.

Our meta-analytical findings have limitations. Most of the included studies were retrospective cohorts; however, updated preprints of placebo-controlled trials solidified our findings. There was a lack of diagnostic criteria for severe COVID-19 between all studies that led to a difference in mechanical ventilation outcomes between the treatment and control groups. The inflammatory markers reduced ventilatory support requirements, and radiological improvement signs were not always presented in the included studies.

## 5. Recommendations 

All placebo-controlled trials must ascertain the optimal dosage of tocilizumab and the potential utility of multiple dosages. IL-6 serum concentration tests must be made routinely available when tocilizumab response is to be assessed [12]. Drug administration in the acute care setting ought to be guided by strict institutional criteria, thus being completely standardized. In addition to the regular follow up period of 28 days, the full course of hospitalization ought to be determined to characterize long-term sequelae in treatment and control groups. 

## 6. Conclusion 

Anti-IL-6 drugs for COVID-19 are a cause of contention since the outbreak of the global pandemic. Tocilizumab, which has had mixed results in RCTs, is being utilized off-label and as an experimental therapy for patients with COVID-19 who are sick or deteriorating with a slight chance of recovery. The ongoing pandemic has created ethical challenges concerning the nonapproved use among patients and choosing the most appropriate patient to receive the experimental therapy in the setting of ongoing randomized controlled trials [21]. The review attempted to link the key tocilizumab studies, find benefits in case of mortality and the risk of mechanical ventilation by the end of treatment, to steer the narrative for ongoing registered trials, which will ultimately form the fate for the use of tocilizumab in COVID-19. 

## Funding

We obtained no funding for this study.
